# Study on Pyrolysis–Mechanics–Seepage Behavior of Oil Shale in a Closed System Subject to Real-Time Temperature Variations

**DOI:** 10.3390/ma15155368

**Published:** 2022-08-04

**Authors:** Lei Wang, Jianzheng Su, Dong Yang

**Affiliations:** 1 State Key Laboratory of Shale Oil and Gas Enrichment Mechanisms and Effective Development, Beijing 100083, China; 2 State Center for Research and Development of Oil Shale Exploitation, Beijing 100083, China; 3 Sinopec Petroleum Exploration and Production Research Institute, Beijing 100083, China; 4 Key Laboratory of In Situ Property Improving Mining of Ministry of Education, Taiyuan University of Technology, Taiyuan 030024, China

**Keywords:** in situ stress, real-time temperature, dynamics, hydrocarbon generation, seepage

## Abstract

In situ mining is a practical and feasible technology for extracting oil shale. However, the extracted oil shale is subject to formation stress. This study systematically investigates the pyrolysis–mechanics–seepage problems of oil shale exploitation, which are subject to thermomechanical coupling using a thermal simulation experimental device representing a closed system, high-temperature rock mechanics testing system, and high-temperature triaxial permeability testing device. The results reveal the following. (i) The yield of gaseous hydrocarbon in the closed system increases throughout the pyrolysis reaction. Due to secondary cracking, the production of light and heavy hydrocarbon components first increases, and then decreases during the pyrolysis reaction. The parallel first-order reaction kinetic model shows a good fit with the pyrolysis and hydrocarbon generation processes of oil shale. With increasing temperature, the hydrocarbon generation conversion rate gradually increases, and the uniaxial compressive strength of oil shale was found to initially decrease and then increase. The compressive strength was the lowest at 400 °C, and the conversion rate of hydrocarbon formation gradually increased. The transformation of kaolinite into metakaolinite at high temperatures is the primary reason for the increase in compressive strength of oil shale at 400–600 °C. (ii) When the temperature is between 20 and 400 °C, the magnitude of oil shale permeability under stress is small (~10^−2^ md). When the temperature exceeds 400 °C, the permeability of the oil shale is large, and it decreases approximately linearly with increasing pore pressure, which is attributed to the joint action of the gas slippage effect, adsorption effect, and effective stress. The results of this research provide a basis for high efficiency in situ exploitation of oil shale.

## 1. Introduction

The abundant oil shale resources have great potential for development and utilization. The quantity of heat energy obtained via the conversion of oil shale is second only to coal in the group of fossil fuels [1,2]. Similar to natural oil, shale oil can be obtained from oil shale by retorting, and it is used as a supplement to natural oil. Oil shale mining methods can be divided into underground (open pit) and in situ mining [3,4]. Oil shale ore must be extracted using underground mining, and then crushed and screened on the ground; it is then subjected to oxygen-free retorting in a ground retort furnace. However, only a minority of the produced shale oil is obtained via underground mining [5,6], and this process is restricted, owing to its negative aspects, such as high costs and serious pollution threats. The in situ mining technology requires drilling holes and arranging wells in the ground. The heat injection well method directly heated the ore body, and the oil and gas formed via the pyrolysis of organic matter were discharged from the production wells; this method ensures environmental and ecological protections, as well as high economic efficiency.

Oil shale deformation, fracture, and oil–gas migration are constrained by in situ stress. The release characteristics of oil and gas and evolution of percolation channels during oil shale pyrolysis subjected to in situ stress require investigation; oil and gas production depend on the pyrolysis temperature. Sadiki et al. [7] reported that the optimal pyrolysis temperature of Moroccan oil shale is 520–630 °C, and they suggested that a large quantity of low-sulfur oil can be obtained within this temperature range. Yu et al. [8] found that increasing the heating rate can effectively improve the pyrolysis yield. Lai et al. [9,10] found that the temperature range in which a large amount of shale oil is produced is 350–460 °C, and the initial production of shale oil predominantly affords light oil, followed by a large quantity of heavy oil. Wang et al. [11] studied the effect of the final pyrolysis temperature on the properties of tar obtained via oil shale retorting. Their study revealed that increasing the final pyrolysis temperature causes the fracture of long-chain aliphatic hydrocarbons into short-chain aliphatic hydrocarbons, and the final pyrolysis improves the quality of shale oil. The above research investigated the optimal oil and gas extraction conditions for oil shale pyrolysis under direct retorting. However, in these studies, the oil shale is in its free state; thus, the pyrolysis tests were conducted in open or semi-open systems.

There is a trend of extracting shale oil from increased depths; as investigations are performed further underground, the rock environment gradually changes from normal temperatures and low stress levels to high-temperature and high-pressure conditions. High temperature conditions considerably affect the mechanical properties of rocks. Suo et al. [12] conducted uniaxial compression tests on shale treated at different temperatures. Their results showed that, with increasing temperature, the compressive strength and brittleness index of the shale decreased. Gautam et al. [13] studied the mechanical properties of sandstone under different temperatures, ranging from room temperature to 900 °C. They found that temperature reduces the adhesion between mineral particles, decreasing the strength and enhancing the ductility of the rock. Zhao et al. [14] studied the mechanical properties of oil shale after high-temperature treatment. They found that the compressive strength of the oil shale decreased with increasing temperature. Wang et al. [15] studied the dependence of the compressive strength of oil shale in both the vertical and parallel bedding directions on the temperature. They discovered that the compressive strength of oil shale in the vertical bedding direction first decreases and then increases with increasing temperature, whereas the compressive strength in the parallel bedding direction monotonically decreases with increasing temperature. However, the changes in the mechanical properties of rock after different temperature treatments do not accurately reflect the mechanical properties of oil shale in the in situ states.

**Figure 1:**
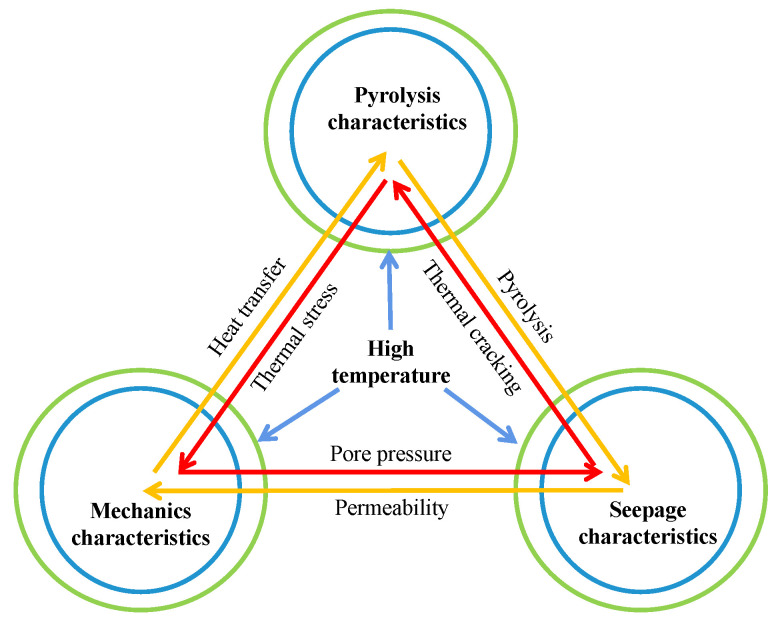
Solid fluid thermochemical multifield coupling process.

In situ mining of oil shale using heat injection is a solid fluid thermochemical multifield coupling process. Additionally, mechanical properties, seepage characteristics, and pyrolysis characteristics are important factors affecting the implementation of in situ production technology. Figure 1 shows these relations. This study uses a thermal simulation experimental device of a closed system to simulate the dynamic behavior of oil shale pyrolysis generating oil and gas, based on the in situ mining technology of oil shale. The compressive strength and permeability of oil shale at real-time high temperatures are investigated using high-temperature uniaxial compression and high-temperature triaxial permeability test systems, respectively. The pyrolysis–mechanics–seepage response laws of oil shale are studied in closed systems and high temperatures. This study provides a theoretical premise for the application of oil shale in situ mining technology.

## 2. Methods

### 2.1. Closed, High-Temperature, and High-Pressure Thermal Simulations

The experimental shale sample was obtained from Fushun, Liaoning, China. Table 1 summarizes the results of oil shale low-temperature retorting.

**Figure 2:**
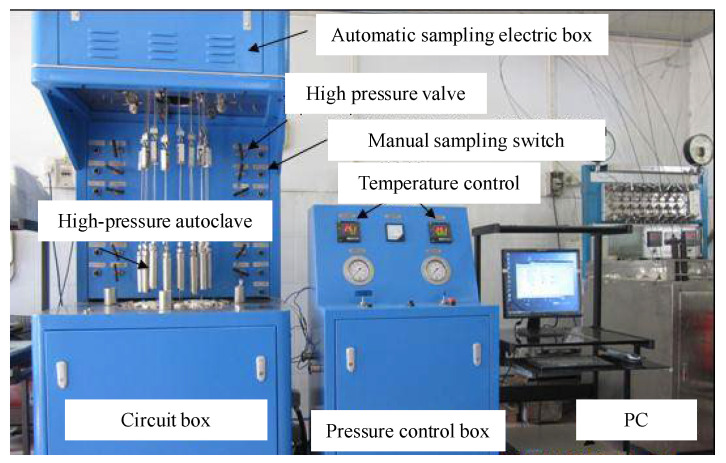
Thermal simulation experimental setup of the closed system.

The thermal simulation experimental device of the closed system (Figure 2) includes an autoclave, vacuum glass tube, high-temperature and high-pressure hydrothermal system, small volume sealing simulation device, gold tube limiting system, and high-temperature and high-pressure diamond pressure chamber microimage hydrocarbon generation simulation system. The autoclave is a cylindrical vessel with a diameter and height of 50 and 100 mm, respectively. The temperature fluctuation of the autoclave is <1 °C, and the pressure is 5 MPa. The sample was sealed in a gold tube (Φ5.5 × 60 mm) under argon atmosphere and placed in the autoclave. The autoclave was then filled with water using a high-pressure pump. The high-pressure water caused the deformation of the gold tube, thus exerting pressure on the sample. The samples were heated at 20–600 °C, at heating rates of 2 and 20 °C/min.

The gold tube containing the sample was removed from the autoclave after heating. The test components included gas (C1–C5), light hydrocarbons (C6–C14), and heavy hydrocarbons (C14+). The gold tube was kept in the vacuum system to release the gas into it. The vacuum system was connected to a chromatography machine to complete the analysis of the C1–C5 hydrocarbon and nonhydrocarbon gases. After the gas analysis, the sample bottle was frozen using liquid nitrogen, in order to collect the liquid hydrocarbon that had diffused into the vacuum glass tube. After removing the sample bottle, the methylene chloride solvent was quickly injected. The gold tube was subsequently kept in the same sample bottle, which was vibrated ultrasonically to accelerate the extraction process. When the vibration time was 1 min, yellow and dark liquid hydrocarbons were observed on the inner walls of the gold tube, and the sample bottle was completely dissolved in the solvent. Thus, the extracted substances were analyzed using chromatography. According to the experimental data obtained for the closed system, the kinetic parameters of the hydrocarbon generation of organic matter in the closed system could be calibrated.

### 2.2. Pyrolysis Reaction Kinetics Analysis Software

**Figure 3:**
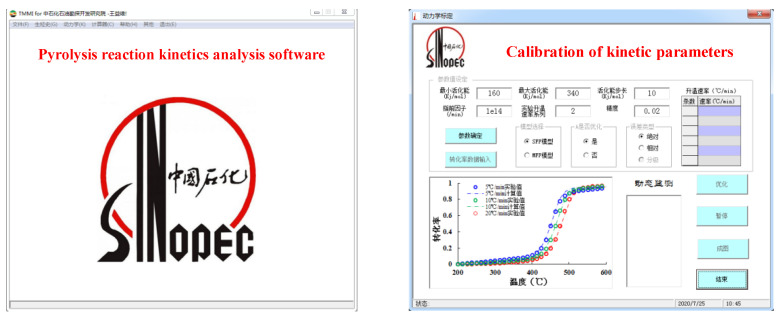
Kinetic software of the in situ pyrolysis of oil shale.

The chemical kinetic models [16,17,18] used to describe the hydrocarbon generation of the organic matter included a general contracting reaction, parallel reaction, serial reaction, and other reaction rate models; each model could be divided into several subtypes. The in situ pyrolysis kinetics software of oil shale (Figure 3) was used to optimize and obtain the kinetic parameters of oil shale pyrolysis using different models and calibration methods. The kinetic parameters include the reaction fraction of organic matter into different gaseous and liquid hydrocarbons. When calibrating the kinetic parameters of hydrocarbon generation, it is necessary to set the range of activation energy (usually 150–340 kJ/mol), step size (generally 10 kJ/mol), and pre-exponential factor.

### 2.3. Real-Time Uniaxial Compressive Strength Tests on Oil Shale at High Temperatures

**Figure 4:**
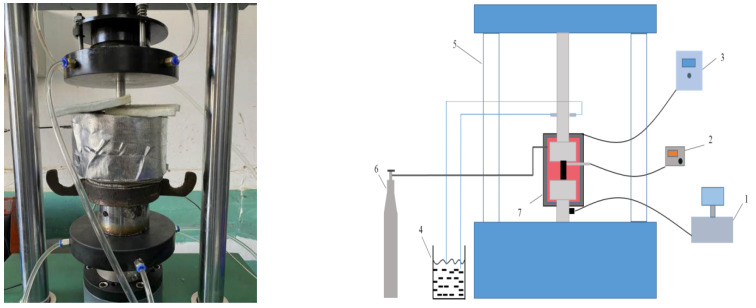
Equipment setup (1. data acquisition system; 2. temperature measurement system; 3. temperature control system; 4. cooling system; 5. universal testing machine; 6. nitrogen bottle; 7. electric heater).

The real-time high-temperature rock mechanical testing system (Figure 4) comprises a loading device and a heating device. The loading device was a computer-controlled, electronic universal testing machine; the test force measurement resolution was 0.005% FS, and the displacement resolution was 0.025 μm. The heating device used the radiation heating method for heating the sample, and the maximum heating temperature of the device was 800 °C; the possible heating rates were 0.1–10 °C/min, and the temperature control accuracy was ±0.1 °C.

Owing to the heterogeneity of the oil shale, the compressive strength of three samples was tested at each temperature to ensure the reliability of the test results. The specific experimental steps used were as follows.

(1) The prepared oil shale sample was placed in the heating furnace, ensuring that the specimen was in the center of the loading head. A 500-N load was then used for prestressing the specimen.

(2) Before heating the specimen, nitrogen was injected into the heating furnace as a protective gas to create an oxygen-free environment. The inlet flow rate of nitrogen was 0.05 L/min.

(3) After the above-mentioned steps were completed, the sample was slowly heated to the target temperature at a heating rate of 1 °C/min. When the temperature in the furnace reached the target temperature, this temperature was maintained in the furnace. The sample was maintained target temperature for 2 h to ensure that the internal temperature of the sample had reached a uniform state.

(4) The loading system was then used to load the oil shale sample at a constant displacement rate of 0.02 mm/min, until the specimen was damaged.

(5) The broken samples were then removed after the test. The furnace was cooled to room temperature, and steps one to four were repeated to test all the samples.

### 2.4. High-Temperature, Triaxial Permeability Test of Oil Shale

**Figure 5:**
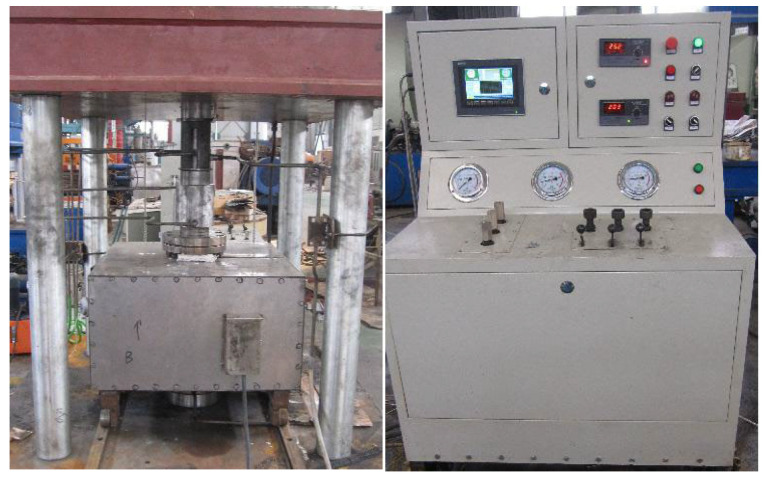
High-temperature triaxial penetration test device.

The high-temperature, triaxial penetration test device (Figure 5) comprises a high-temperature and high-pressure reactor, hydraulic control system, seepage test equipment, and temperature control system. The permeable medium used was high-purity nitrogen. The simulated burial depth was 150 m, stress gradient was 0.025 MPa/m, and lateral pressure coefficient was 1.2. The pore pressures considered were 1, 1.5, and 2 MPa.

The following procedure was used to test the specific permeability of the samples.

(1) The sample was sealed using a red copper sleeve and placed in the reactor; the air tightness of the entire equipment was tested simultaneously.

(2) The axial pressure and confining pressure were applied alternately to the preset values, maintaining a constant pressure. The sample was slowly heated using an external heating mantle at a heating rate of 0.1 °C/min.

(3) The temperature was raised to 100 °C and then kept constant for 1 h to conduct permeability tests under various pore pressures. The temperature was then raised in increments of 100 °C, and permeability testing was conducted until the temperature reached 600 °C.

(4) According to Darcy’s law [19,20], the permeability of oil shale at different temperatures and pore pressures can be calculated using the following expression:

(1) $$k=\frac{2Qp_{0}L\mu }{\left(P_{1}^{2}-P_{2}^{2}\right)A},$$

where *k* denotes the permeability of oil shale [m^2^], *Q* represents the volume flow rate of nitrogen [m^3^/s], *P*_0_ denotes atmospheric pressure, 0.1 MPa, *L* represents the sample length [m], *μ* denotes the dynamic viscosity of the gas [MPa s], *P*_1_ represents the pressure at the inlet end of the sample [MPa], *P*_2_ denotes the pressure at the outlet end of the sample [MPa], and *A* represents the cross-sectional area of the sample [m^2^].

## 3. Results and Discussion

### 3.1. Regularity of the Formation of Hydrocarbon Products from the Pyrolysis of Oil Shale

The production law of oil and gas from oil shale pyrolysis at different heating rates was very similar. The yield and conversion obtained at a heating rate of 2 °C/min were higher than those obtained at a heating rate of 20 °C/min. Therefore, the characteristics of the pyrolysis output conducted using a heating rate of 2 °C/min were studied.

**Figure 6:**
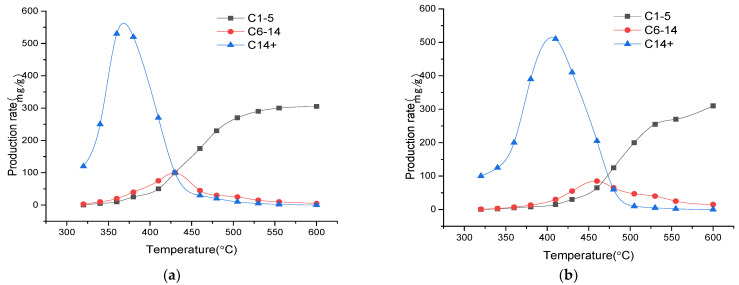
Yield curves for each component in the closed system; (**a**) 2 °C/min; (**b**) 20 °C/min.

Figure 6 shows the change in the composition of the pyrolysis products of oil shale in the closed system. Owing to its low activation energy, the heavy oil component accounts for a large proportion during the initial stage, and the yield of heavy components gradually decreases, due to secondary cracking processes in the later stage. In the closed system, the overall yield of gaseous hydrocarbons increased as the reaction initially proceeded; this yield only decreased slightly during the last stage of the reaction. In the last stage, the number of reactants was no longer sufficient, which decreased the reaction rate. The yields of light and heavy oil showed an initial increase, followed by a decrease; this decrease was because of the secondary cracking of the products. Light oil was mainly cracked into gaseous hydrocarbons, whereas heavy oil was mainly cracked into light oil and gaseous hydrocarbons. Heavy oil could start secondary cracking at a lower temperature than the light oil because, at the initial stage of secondary cracking, heavy oil is frequently cracked into light oil, and the reaction activation energy for this process is low.

The yield curves of the three components show that the gas generation process lasts the longest, and the gas is generated from the beginning to the end of the experiment. The generation duration of light oil components is relatively short, and the yield reduced to a low level before the temperature of ~550 °C was reached. The time period during which heavy oil components are generated is the shortest, and hydrocarbon generation ends at 500 °C.

### 3.2. Kinetic Analysis of Hydrocarbon Generation via Oil Shale Pyrolysis

Kerogen is a complex macromolecule with multiple functional groups and bond types. Its source, composition, structure, and bond types are extremely complex. The parallel reaction model can accurately capture the kinetic essence of the hydrocarbon generation process of kerogen. The calibration of the parallel non-first-order reaction model is difficult, and the hydrocarbon generation process of sedimentary organic matter can be described using the parallel first-order reaction [21,22]. Therefore, the first-order reaction is typically used when parallel reactions are used to study the hydrocarbon generation process of organic matter. It is assumed that the activation energy distribution function of the parallel reaction obeys the discrete distribution. The activation energy is distributed at certain intervals, across a certain range, using the discrete distribution. The following equation can describe the parallel first-order dynamic model [23]:

(2) $$x=\int_{0}^{\infty }\exp[-\int_{0}^{t}k(T)dt]D(E)dE.$$

**Figure 7:**
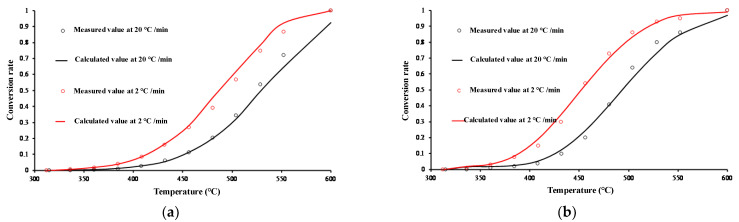
Dynamic fitting effect of gas hydrocarbons generated using pyrolysis of oil shale. (**a**) CH_4_; (**b**) C_1–5_.

Figure 7 shows the fitting of the parallel first-order reaction kinetic model for the hydrocarbon generation using oil shale pyrolysis in the closed system. With increasing temperature, the hydrocarbon generation conversion rate gradually increases; the lower the heating rate, the higher the hydrocarbon generation conversion rate for a given temperature. This reflects the time dependence of the hydrocarbon generation rate, which is in agreement with the theory of compensation effect of time on temperature in the process of hydrocarbon generation from organic matter. The distribution of activation energies in the hydrocarbon generation reaction can be relatively concentrated. In addition, the chemical kinetic model fits well with the experimental conversion rates of organic matter into oil and gas at different heating rates and temperatures, thus indicating the feasibility of the selected model. Table 2 presents the kinetic parameters of the hydrocarbon generation reaction using oil shale pyrolysis. The pre-exponential factor of the oil shale pyrolysis in the closed system is 10^16^ min^−1^. With the increase in activation energy, the reaction fraction of oil shale pyrolysis to hydrocarbon first increases and then decreases. When the activation energy is between 210 and 280 kJ/mol, the reaction fraction is significant, and activation energies are concentrated and have a clear primary peak. Owing to the complexity of the organic matter composition, the activation energies will have a certain distribution range, although they tend to be dominated by some bond types, while the content of other bond types is low, which can be ignored.

### 3.3. Real-Time Evolution of the Compressive Strength of Oil Shale at High Temperatures

**Figure 8:**
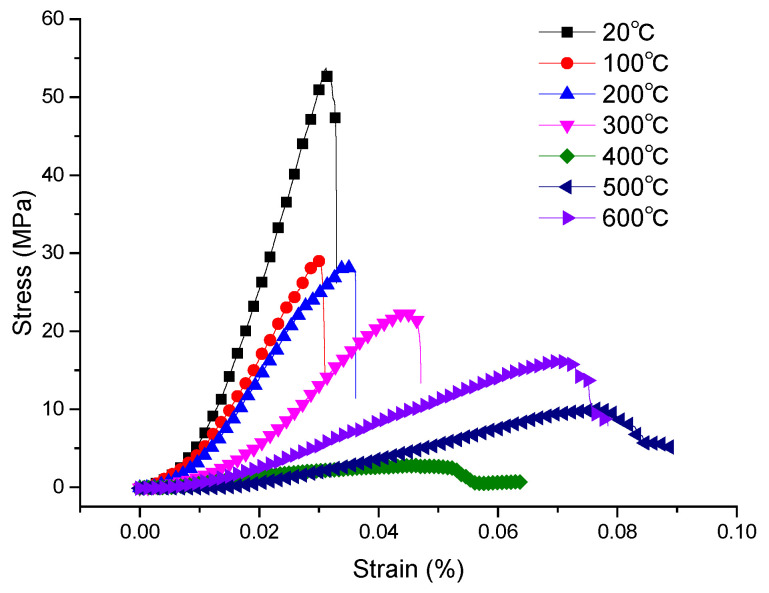
Stress–strain curve of oil shale at high temperatures.

To study the influence of temperature on the uniaxial mechanical properties of oil shale, the stress–strain curves of representative specimens at different temperatures were obtained (Figure 8). In general, according to the trend of compressive strength changing with temperature, it can be divided into two stages. Phase I: as the temperature increases from 20 to 400 °C, the peak load and slope of the stress–strain curve of oil shale decrease while the peak strain increases. Phase II: in the range of 400–600 °C, the peak load, curve slope and peak strain of the stress–strain curve increase. Therefore, 400 °C can be regarded as the threshold temperature at which the behavior of the compressive strength changes.

The shapes of the stress–strain curves at different temperatures are similar; in general, the curves can be divided into four stages, as follows.

(1) The compaction stage: In this stage, the primary change is that some low strength pores and fractures are closed due to the external load. With increasing load, the pores/fractures are gradually compressed.

(2) The linear elastic stage: In this stage, a linear relationship exists between stress and strain. Because the internal stress in oil shale is relatively low, the pore/fracture structures do not expand, and the mineral particles in oil shale are subject to recoverable elastic deformation.

(3) The yield stage: In this stage, the stress–strain curve transitions to show nonlinear behavior; with increasing stress, the stress–strain curve begins to form a plateau. The reason for this phenomenon is that, when the internal loads within the oil shale exceed a certain limit, its internal fractures will expand, and mineral particles will become more distant, thus resulting in the continuous increase in the number and size of internal microfractures, a continuous breaking of internal stress balance, and a continuous consumption of accumulated energy [24,25].

(4) The failure stage: In this stage, the internal fractures expand rapidly with the continuous increase in stress. When the peak stress is reached, the stress–strain curve disappears or remains at a residual strength level.

**Figure 9:**
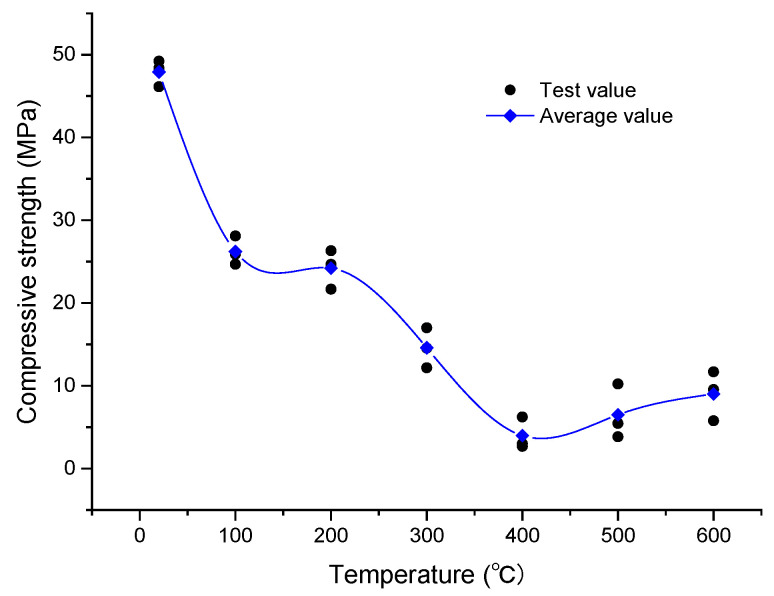
Variation in the compressive strength of oil shale with increasing temperature.

Figure 9 shows the variation in oil shale compressive strength for temperatures between 20 and 600 °C. With increasing temperature, the compressive strength of oil shale can be roughly divided into two phases.

Phase I (20–400 °C): In this phase, the compressive strength decreases considerably with increasing temperature. The compressive strength of oil shale at room temperature is 47.9 MPa. At temperatures of 100 and 200 °C, the compressive strength decreases to 26.22 and 24.21 MPa, respectively; thus, at these temperatures, the compressive strength of oil shale is less than half of its compressive strength at room temperature. During the heating process, the large fractures will first expand along the bedding, thus decreasing the cohesion between the internal matrix of the oil shale and different bedding planes; this induces a rapid decrease in the compressive strength. When the temperature increases from 200 to 400 °C, the compressive strength decreases to 3.97 MPa. In this temperature range, kerogen transforms from a solid to a liquid form and then decomposes into asphaltene. Oil shale has a certain number of open pores and fractures; this structure permits asphaltene to migrate and fill the existing fractures. However, asphaltene will decompose into shale oil and mixed hydrocarbon gas, which will increase the internal pressure of pores and promote the rapid expansion of pore-fracture structures. Overall, it shows the damage to the mechanical strength of the rock.

Phase II (400–600 °C): In this phase, the compressive strength increases slowly with temperature. The compressive strength increases to 9.0 MPa at 600 °C. At this temperature, the pyrolysis of oil shale is largely complete. Kaolinite loses its crystal water and turns into metakaolinite [26,27] at 520–540 °C, which increases the compressive strength.

### 3.4. Evolution of Oil Shale Permeability with Increasing Temperatures

**Figure 10:**
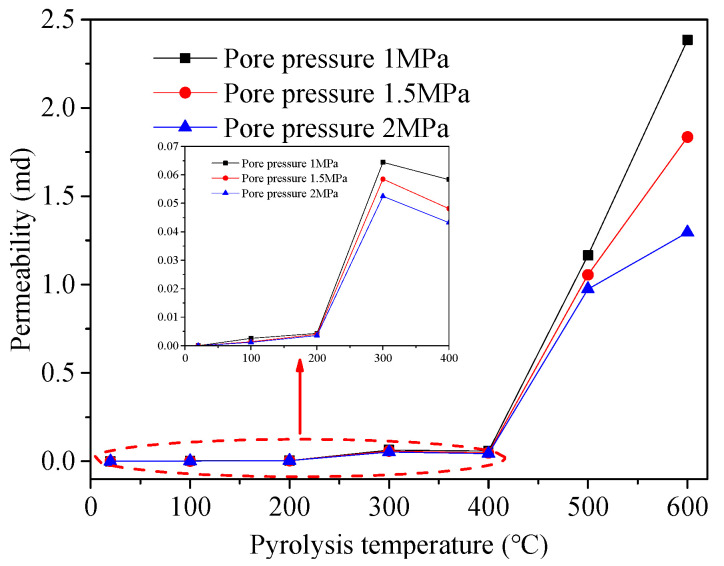
Correlation between permeability and temperature.

Figure 10 shows the influence of the pyrolysis temperature on the oil shale permeability. At 100–200 °C, the oil shale permeability is in the order of 10^−3^ md. When the temperature is in the range 300–400 °C, the permeability of oil shale is in the order of 10^−2^ md. When the temperature exceeds 500 °C, the permeability of oil shale is found to be large. The process of the change can be divided into three stages, according to the rate of change of the permeability with temperature.

The first stage: When the temperature increases from 20 to 300 °C, the permeability remains low; in this stage, the rate of increase in the permeability with increasing temperature is low. In this temperature range, the organic matter is not effectively pyrolyzed, and the increase in the permeability is mainly attributed to the thermal cracking induced by thermal stress in the rock mass. Generally, the degree of thermal cracking in oil shale at low temperatures is also relatively low.

The second stage: When the temperature increases from 300 to 400 °C, the permeability of the oil shale decreases. In this temperature range, the degree of thermal cracking in the oil shale increases, but kerogen also forms viscous primary products similar to tar and asphalt during pyrolysis, thus filling the space in the pores and fractures.

The third stage: When the temperature increases from 400 to 600 °C, the permeability increases considerably. A large amount of organic matter decomposes to form oil and gas; thus, pores and fractures are formed in the rock mass. Moreover, the release rate of high-temperature oil and gas is high. The opening of pores and fractures further increases during the migration and drainage of oil and gas. At the same time, the degree of thermal cracking of the rock mass at high temperature increases, thus inducing a sharp increase in the permeability of the oil shale.

**Figure 11:**
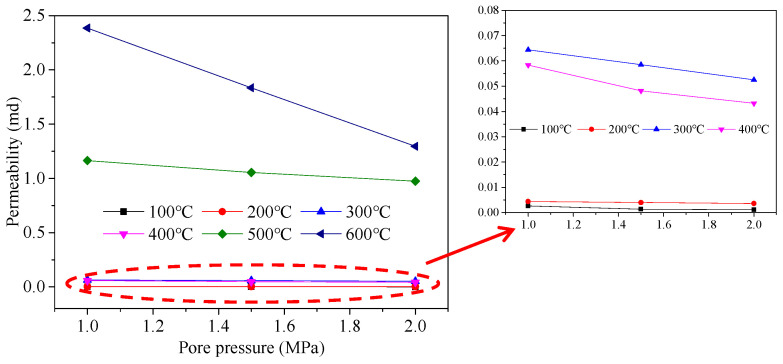
Correlation between permeability and pore pressure.

Figure 11 shows the influence of pore pressure on the permeability of oil shale. The permeability of the oil shale decreases approximately linearly with increases in the pore pressure. The permeability test medium used in this test was high-purity nitrogen. This phenomenon is primarily caused by the influences of the gas slippage and adsorption effects, as well as effective stress [28,29,30] (which is approximately equal to the difference between the volume stress and pore pressure). The density of the injected nitrogen increases with increasing injection pressure, and the slippage effect is less obvious. However, higher injection pressures lead to larger gas flow rates, and the adsorption of a gas using the mineral matrix and framework in the oil shale is strengthened. This phenomenon decreases the permeability of the sample. However, when the pore pressure increases, the effective stress decreases, the opening of pores and fractures within the oil shale increases, and the seepage path widens; these effects increase permeability.

## 4. Conclusions

The formation and migration of the pyrolysis products in the in situ mining of oil shale are affected by in situ stresses and pyrolysis temperatures. In this study, the pyrolysis–mechanics–seepage behavior of oil shale in a closed system has been investigated at various pyrolysis temperatures. The main conclusions are as follows.

(1) In the closed system, the yield of gaseous hydrocarbon from oil shale pyrolysis shows an increasing trend, while the yields of light and heavy oil first increase and then decrease. The yield of light oil components decreases to a low level at ~550 °C.

(2) The parallel first-order reaction kinetic model is a good fit for the observed pyrolysis and hydrocarbon generation processes of oil shale. With increasing temperature, hydrocarbon generation conversion rate increases gradually, and the distribution of activation energies of hydrocarbon generation reactions is relatively concentrated, with a clear primary peak. The average activation energies of methane and gas from oil shale pyrolysis are 257 and 246 kJ/mol, respectively.

(3) The uniaxial compressive strength of oil shale can be divided into two phases. The first phase is denoted by the temperature range of 20–400 °C, where a considerable decrease in compressive strength is observed. The second phase is denoted by the temperature range of 400–600 °C, where an increase in the compressive strength is observed. At 400 and 600 °C, the compressive strengths are found to be 3.97 and 9.0 MPa, respectively. The temperature 400 °C can be regarded as the threshold temperature, as it denotes a sudden change in the compressive strength.

(4) When the temperature is between 20 and 400 °C, the oil shale permeability is in the order of 10^−2^ md. When the temperature is higher than 400 °C, the permeability of oil shale is larger. The permeability decreases approximately linearly, thus increasing the pore pressure. This is hypothesized to be induced by the combined effect of gas slippage, adsorption, and effective stress.

## Figures and Tables

**Table 1:** Fischer assay analyses of the Fushun oil shale.

Fischer Assay Analysis (wt.%, ad)	Composition (%)
Oil yield	8.07
Water yield	1.78
Residue	86.73
Gas + loss	3.42

**Table 2:** Kinetic parameters of hydrocarbon generation during oil shale pyrolysis.

Activation Energy (kJ/mol)	The Pre-Exponential Factor (min^−1^)	Reaction Fraction of Organic Matter to Methane	Reaction Fraction of Organic Matter to Gas
150	10^16^	5.38 × 10^−4^	4.35 × 10^−3^
160		5.38 × 10^−4^	4.35 × 10^−3^
170		5.38 × 10^−4^	4.35 × 10^−3^
180		5.27 × 10^−4^	4.34 × 10^−3^
190		6.80 × 10^−4^	5.64 × 10^−5^
200		1.64 × 10^−4^	1.77 × 10^−4^
210		1.75 × 10^−2^	1.32 × 10^−7^
220		8.57 × 10^−3^	6.64 × 10^−2^
230		8.92 × 10^−2^	1.64 × 10^−1^
240		7.45 × 10^−2^	2.31 × 10^−1^
250		2.68 × 10^−1^	2.26 × 10^−1^
260		1.51 × 10^−1^	1.64 × 10^−1^
270		2.67 × 10^−1^	9.29 × 10^−2^
280		1.21 × 10^−1^	2.71 × 10^−2^
290		6.89 × 10^−6^	2.89 × 10^−4^
300		3.18 × 10^−4^	1.64 × 10^−4^
310		2.51 × 10^−5^	2.89 × 10^−4^
320		1.30 × 10^−6^	1.61 × 10^−4^
330		6.13 × 10^−7^	4.90 × 10^−3^
340		5.92 × 10^−6^	5.01 × 10^−3^
Average activation energy (kJ/mol)		256.81	246.13

## Data Availability

The data used to support the findings of this study are available from the corresponding author upon request.
